# How Perfectionism and Eating Disorder Symptoms Contribute to Searching Weight-Loss Information on the Internet?

**DOI:** 10.3390/medicina55100621

**Published:** 2019-09-23

**Authors:** Katarina Prnjak, Ivan Jukic, Anita Lauri Korajlija

**Affiliations:** 1 Translational Health Research Institute (THRI), School of Medicine, Western Sydney University, Sydney 2560, Australia; 2 Sport Performance Research Institute New Zealand, Auckland University of Technology, Auckland 0632, New Zealand; jukazy@gmail.com; 3 Faculty of Humanities and Social Sciences, University of Zagreb, Zagreb 10000, Croatia; alauri@ffzg.hr

**Keywords:** online searching, health, diet, body mass index, women

## Abstract

*Background and Objectives*: Eating disorder (ED) symptoms are a growing problem and modern technologies introduced a new and unexplored potential risk factor for vulnerable individuals. It is fairly common for women to use the Internet in order to find information about various weight-loss methods, but it was further questioned whether perfectionism and eating disorder symptomatology could be linked to this behavior. *Materials and Methods*: Participants were 228 women (Mean age = 30.5; SD = 9.43) recruited via social media, who provided responses on measures of perfectionism, eating disorder symptoms, and a short check-list measuring the frequency of online searching about five topics (food, diet, exercise, body appearance, and eating disorders). *Results*: Hierarchical multiple regression analysis showed that the BMI and Discrepancy subscale of APS-R significantly predicted online searching, along with eating disorder symptomatology. Moreover, mediation analyses resulted in a significant indirect effect, but not a direct effect, indicating that eating disorder symptomatology fully mediated the relationship between BMI and online searching, as well as between maladaptive perfectionism and online searching. *Conclusion*: These findings shed light on a high BMI and maladaptive perfectionism as potential risk factors for eating disorder-related behavior on the Internet. More attention to online-seeking behavior among women symptomatic of ED is warranted, and websites containing such topics should include information about professional help for eating disorder-symptomatic individuals.

## 1. Introduction

In modern society, online information is easily accessible to people all around the world, and most of Internet users seem to be adolescents and emerging adults [1]. Furthermore, people nowadays often use the Internet to search for health information [2] and weight-loss methods [3]. However, information found on the World Wide Web is not always trustworthy and may have an impact on everyday life decisions. For example, the effectiveness of some weight-loss methods is often misleadingly communicated [4] and the Internet users are not always literate when it comes to health information, especially adolescents [5]. Moreover, today, various eating patterns are being promoted on the Internet, aiming at those willing to be healthy and/or lose weight. Nonetheless, following certain advice on nutrition can sometimes provoke severe eating disturbances in those who have some predispositions, and thus reach the level of an eating disorder (ED). It is, therefore, important to observe and control the triggers for these disorders since they impair physical health and psychosocial functioning [6], and some triggers might be related to Internet usage.

It has been found that 15% of current and 35% of lifetime ED patients are prone to problematic Internet usage, defined as uncontrollable, time-consuming, and life-quality impairing behavior [7]. Social media has also become very popular in the latest years and its contribution to body dissatisfaction might be worrisome. For instance, it has been shown that the time spent on the Internet is positively connected with a drive for thinness and body surveillance, while Facebook users also have significantly greater body image concerns [8]. Exposure to pro-ED websites is especially concerning, since it was shown to have an effect on dieting behavior, body dissatisfaction, and negative affect, although not on bulimic symptoms [9]. However, weight-loss advice imposed through the Internet is usually intended for those who struggle with being overweight. Thus, the Internet is considered to be a useful tool for searching for information about weight-loss interventions and programs in the cases of those who need them [10]. However, obese individuals are sometimes prone to relying on quick and unrealistic weight-loss advice presented on certain websites [11] and the underlying problem could be the lack of nutritional knowledge among those who want to lose weight. Additionally, some findings [12] indicate that adolescents with an ED, as well as their parents, do not have enough basic knowledge about nutrition and “healthy” diets.

Since personality traits contribute to the onset and the maintenance of an ED [13], it is important to consider them while investigating this area. The association between perfectionism and ED symptomatology in previous research is well established [14]. Maladaptive perfectionism was often associated with higher psychological distress and was, therefore, associated with various psychopathological conditions, such as depression, anxiety and EDs [15]. Adaptive perfectionism, on the other hand, was shown not to correlate with depressive symptoms nor negative affect [16]. Nevertheless, both adaptive and maladaptive facets seem to be elevated among ED patients, unlike in other psychiatric disorders [14]. For instance, self-oriented perfectionism, which is considered to be adaptive, was a significant predictor of ED symptoms, possibly through the increased evaluation of shape and weight [17]. Furthermore, the commitment to “food rules” has been found to be a mediator between the relationship of self-oriented perfectionism and eating pathology [18]. Results of these and many other studies [19] indicate that adaptive and maladaptive perfectionism are the risk factors for ED pathology and that personal weight goals might play a greater role in the development of an ED, rather than socio-cultural expectations. Additionally, since perfectionistic tendencies seem to be associated with higher motivation for goal achievement [17], both adaptive and maladaptive perfectionism might contribute to online searching for ED-related topics, if this behavior is a potential indication of pursuing a weight-loss goal. All things considered; it is important to examine which role these two perfectionism dimensions play in online searching behavior that might actually represent a manifestation of unhealthy eating attitudes in online environment.

Finding out which aspect of perfectionism contributes to online searching for these topics might be important if this behavior characterizes individuals with subthreshold ED. Furthermore, age and body mass index (BMI) could be the indicators of seeking ED-related information on the Internet, and it would be beneficial to determine if these might be antecedents of potentially worrisome behavior presented on the Internet. In recent times, online behavior has been attracting scientific attention in general, especially in the area of psychopathology. However, little is known about the relationship between perfectionism and online searching behavior that is potentially accompanied by eating concerns. Therefore, the main aim of the present study was to determine whether adaptive and maladaptive perfectionism, alongside ED symptoms, explain the online searching for ED-related topics. Answering some of the previous questions could lead to a better understanding of behavior that might be concerning in an ED context, as well as suggestions of potential modifications of online environments that might trigger some eating disturbances. We hypothesize that perfectionism will be a contributing factor to searching ED-related terminology on the Internet, due to its evident relationship with ED symptomatology, while the relationship between other variables was of an exploratory nature.

## 2. Materials and Methods

### 2.1. Participants

Two-hundred-twenty-eight women from 17 to 66 years of age (M = 30.5; SD = 9.43) participated in this study. Inclusion criteria for participation in this study was to be a woman above 16 years of age (as parental approval would have to be obtained in the case of younger participants). Those respondents who stated that had never used the Internet for searching information about diet, exercise, and body appearance were automatically excluded from the study. Most of the participants had a high-school degree (32.5%), while 29.4% of them had a Master’s degree. Fifty-one percent of the participants were employed and 32% were students at the time of data collection. Most of the subjects reported being in a relationship (35.1%), as well as being married (33.3%). Thirty-seven percent of participants reported current dieting behavior, while 6% had an ED diagnosis at the time of data collection. The Ethics approval code is 1-ED-PSI-09022018.

### 2.2. Measures

The SCOFF questionnaire (Sick, Control, One Stone, Fat, Food; Morgan, Reid, & Lacey, 1999 [20]) was used for the selection of individuals with a higher risk of development of an ED. SCOFF consists of five questions with yes/no answers. Questions are related to the baseline symptoms of ED, such as compensational behavior, feeling of losing control, sudden weight-loss, distorted body image, and food preoccupation. If the answer to at least two questions is positive, it is likely that the person has an ED [21]. This questionnaire by itself cannot serve for diagnostic purposes due to the possibility of falsely classifying someone as ED positive, but it is simple and very efficient in recognizing people with ED symptomatology [21]. Cronbach’s alpha coefficient of internal consistency was shown to be from α = 0.47 to α = 0.66 in previous research [22,23,24]. In the current study, exploratory factor analysis yielded a one factor solution which explained 41% of variance. Cronbach’s alpha was 0.63.

The Almost Perfect Scale-Revised (APS-R; [25]) is an instrument measuring perfectionism with 23 items on the 7-point Likert-type scale. APS-R contains three factors: High Standards, Order, and Discrepancy. High Standards (7 items) represents the need for achievement (e.g., I expect the best from myself), Order (4 items) indicates the need for orderliness (e.g., Neatness is important to me.), and Discrepancy (12 items) measures the difference between expected standards and real achievements (e.g., I often feel frustrated because I can’t meet my goals). High Standards and Order are indicators of adaptive perfectionism, while Discrepancy measures maladaptive perfectionism. Internal consistency of the subscales measured as Cronbach’s alpha ranged from 0.73 to 0.93 [26,27,28]. In the present study, exploratory factor analysis yielded a three-factor solution which explained 34.4%, 19.3%, and 10.8% of variance. Cronbach’s alpha for the three subscales were 0.84, 0.88, and 0.94, respectively.

A check-list of topics potentially related to EDs (and of importance to those who might experience eating concerns) was designed for the purposes of this study to assess how frequently participants searched for the following terminology on the Internet: food, diet, exercise, body appearance, and EDs. There were four answering options: Never, rarely, sometimes, and often, which were later coded with numbers 1–4. Each of the five items was accompanied with a question: “Please, write down some of the terms related to this topic that you search online”. Participants could write down terms on a blank line. The frequency of searching each topic were combined into a total result, since exploratory factor analysis yielded a one factor solution which explained 50%. Cronbach’s alpha was 0.75.

### 2.3. Procedure

Researchers received approval from the Ethics Committee of Department of Psychology at the Faculty of Humanities and Social Sciences prior to data collection, which was conducted online using the SurveyMonkey website. Invitation to participate in this research was shared via social media, aiming at a broad range of women older than 16 years of age (who do not warrant a parental permission). On the beginning of survey, general instructions were written, including the name and the e-mail address of researcher. The anonymity of the participants was guaranteed, as well as the possibility to terminate participation at any moment. After giving a formal consent, participants had to fulfill sociodemographic data. Participants then provided their answers on a check-list, APS-R, and SCOFF questionnaire. Overall, the duration of participation lasted approximately 10 min. Participants were not rewarded for their participation in this study.

### 2.4. Statistical Analysis

Person’s correlation coefficients were computed to explore links between the main variables of the study. In order to determine independent contributions to ED-related online searching behavior, a hierarchical regression analysis was carried out in three steps. To test a potential mediating effect of ED symptoms between perfectionism and ED-related seeking terms on the Internet, a mediation was conducted. All statistical analyses were performed using the software package SPSS (IBM SPSS version 24.0, Chicago, IL, USA) with a Process Macro add-on [29] for mediation analysis.

## 3. Results

### 3.1. Descriptive Statistics

The descriptive statistics and Person’s correlation coefficients between the main variables are presented in Table 1. BMI (weight (kg)/height (m^2^)) ranged from 15.57 to 42.58 (M = 23.34, SD = 4.25), which indicates that participants were, on average, of normal weight. However, only 4.4% of them were considered underweight, 18% overweight, and 8.4% obese according to BMI measurements. Furthermore, 43.4% of the participants in the present study were considered at higher risk for an ED development according to SCOFF. Regarding online searching for topics, participants most commonly reported searching information about macronutrients, calories, as well as various exercise types.

### 3.2. Hierarchical Regression Analysis

The assumption of multicollinearity absence was confirmed (VIF < 5; tolerance > 0.2; [30]. Searching for ED-related topics on the Internet was considered as a behavioral outcome, perfectionism as a stable personality trait [31], and ED symptoms as symptoms of a severe psychiatric condition [6]. Thus, the total score on the check-list for online searching ED-related topics was used as the criterion variable (Table 2). In the first step, the BMI was shown to be a significant predictor (*p* < 0.001) of online searching frequency. Three facets of perfectionism were introduced in the next step, and only the subscale Discrepancy had a significant beta coefficient (*p* = 0.002). Finally, in the third step, the SCOFF score also appeared as the significant predictor (*p* < 0.001). This set of predictors explained a total of 23.7% of online searching variance. In addition, it can be noticed that the beta coefficient of the Discrepancy subscale became insignificant after the SCOFF result was added in the subsequent stage, and thus the mediation test is required to explore the potential mediating effect of the ED symptomatology.

### 3.3. Mediation Analyses

Total, direct, and indirect effects were tested for both Discrepancy and BMI as predictors (Table 3). Total effect was shown to be significant in case of Discrepancy (*p* = 0.008) and BMI (*p* < 0.001), while direct effects were not significant (*p* = 0.726; *p* = 0.142, respectively), meaning that Discrepancy and BMI do not predict online searching frequency alone. Confidence intervals of the indirect effect did not include absolute zero, which indicates that this effect is statistically significant with 95% level of confidence. In addition, the indirect effect appears to be greater in the case of BMI.

## 4. Discussion

**Figure 1:**
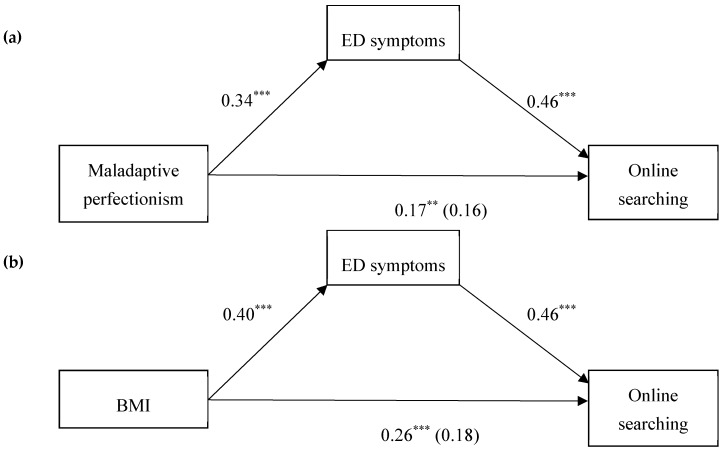
Standardized regression coefficients for the relationship between: (**a**) Maladaptive perfectionism and online searching; (**b**) BMI and online searching, as mediated by ED symptoms. The standardized regression coefficients between: (**a**) Maladaptive perfectionism and online searching; (**b**) BMI and online searching, controlling for ED symptoms, are in the parentheses. BMI, body mass index; ED, eating disorder. ** *p* < 0.01, *** *p* < 0.001.

The findings of this study indicate that maladaptive perfectionism could be a risk factor for online searching about ED-related topics since only Discrepancy, among other perfectionism subscales, successfully predicted online searching for ED-related topics among participants of the current study. Those women who were prone to maladaptive perfectionism more often searched ED-related topics online, while adaptive perfectionism showed no association with searching these topics on the Internet. Furthermore, entering age and BMI in the model demonstrated that BMI was a significant predictor of searching these topics on the Internet. More specifically, searching about topics related to eating concerns is a more common behavior among women who had a higher BMI. In addition, after including ED symptomatology in the final step of hierarchical regression analysis, the contribution of BMI and maladaptive perfectionism became insignificant. Finally, mediation analyses indicated that ED symptoms represented a mediator between BMI and online searching about ED-related topics, as well as maladaptive perfectionism and searching these topics on the Internet (Figure 1). Therefore, BMI and maladaptive perfectionism were not directly connected with online searching behavior because ED symptoms as a mediator explained these relationships to a greater extent.

Forty-three percent of women in the current study were identified as ED symptomatic, and this number seems to be greater than that observed in other studies [22,23]. The possible explanation, due to the fact that 36.6% of participants reported maintaining a diet, is that more food focus and preoccupation was experienced as a consequence of such behavior. Additionally, Sanchez-Armass et al. [32] highlighted the possibility of false-positively classified cases based on SCOFF results, indicating that this questionnaire might have overestimated the exact number of ED symptomatic women in our sample. Moreover, correlation analysis showed a positive association between age and ED symptoms, meaning that older women in this sample experienced more ED symptoms. This is in accordance with what Mangweth-Matzek, Hoek, and Pope Jr [33] reported in their review, that ED pathology has been found in older women, who more frequently reported a binge eating disorder and subthreshold EDs than the diagnosis of anorexia and bulimia. However, age was not an independent predictor of online searching behavior, probably due to overlapping with BMI variables in the first model. 

BMI was a significant predictor of online searching for ED-related topics in the present study, indicating that women who have higher BMI more often searched for these topics on the Internet. According to this measure, 26.4% of women in the current study were either overweight or obese, which suggests that these women were more prone to experiencing symptoms of binge eating disorder or bulimia nervosa rather than anorexia nervosa, since anorexia diagnosis requires a low BMI [6]. Notwithstanding, the relationship between the BMI and online searching was fully mediated by ED symptomatology, suggesting that women with a higher BMI experienced more ED symptoms and consequently reported more frequent searching for ED-related terms on the Internet. In spite of many studies [9,34] investigating the role of pro-ED websites on ED-symptomatic individuals, the present study sought to prove that a broader spectrum of Internet sites potentially plays an important role for ED symptomatic individuals. Furthermore, women with symptoms of binge eating disorder or bulimia might be those who spend more time searching for weight-loss methods on the Internet than women affected with anorexia nervosa.

Lehmann and Konstam [35] reported a positive correlation between maladaptive perfectionism and problematic internet usage. Similarly, the same relationship was yielded in a current study, but maladaptive perfectionism was not directly associated with online searching behavior, rather as a mediator. Maladaptive perfectionism hence could be seen as a vulnerability factor for ED symptoms, and consequently for increased ED-related searching on the Internet. It might be that those women who scored higher on the maladaptive perfectionism scale experienced some ED symptoms, since previously established findings indicate that maladaptive perfectionism is positively associated with ED symptoms [36]. Bardone-Cone et al. [14] also mentioned that, if desired outcomes are unmet, increase in negative affect and aversive self-awareness might occur. Consequently, this can lead to binge eating as a way of escape [14]. Also, some findings indicate that worry about imperfection is, among other types of perfectionism, the most significant predictor of unhealthy eating behavior [37]. Therefore, the findings of the present study confirmed the idea that maladaptive perfectionism operates as a vulnerability factor for many psychiatric disorders, or in this case, ED.

Findings of the present study suggest that experiencing ED symptoms might induce more frequent searching for topics related to EDs on the Internet. Schroeder [34] also reported that female patients with ongoing ED treatment believe that visiting pro-ED websites had worsened their symptoms and encouraged some common eating-related obsessions. Furthermore, one meta-analysis demonstrated a small to moderate effect of these websites on ED symptoms [9]. Hence, these findings indicate that websites containing pro-ED content are considered harmful for patients in ongoing treatment. However, not all websites are classified as pro-ED, and other online sites might be important for those having eating concerns. For instance, websites that promote certain diets and weight-loss methods might also contribute to the rise and/or maintenance of unhealthy feeding habits or eating preoccupations. Even though online places that provide users with nutritional information about food are generally useful and harmless, in the case of ED symptomatic individuals, these information might gain a bigger emphasis and influence their eating behavior in a negative way. Thus, it might be useful for such pages to include information about the severity of EDs as psychiatric conditions, and description of symptoms often experienced in disease onset as well. In addition, since ED symptomatology could induce more frequent online searching, such websites could provide the visitors with advice about seeking professional help. Finally, practical implications should aim at the clinicians who work with ED patients. They should pay more attention to the patients’ behavior on the Internet since the frequency of its usage seems to be connected with ED symptomatology. For example, clinicians could ask patients how often they visit websites that contain topics such as diets and exercise and inspect potential reasons for such behavior.

There are some limitations of this study that need to be addressed. First, ED symptomatology is a variable with only six possible results due to the brevity of the SCOFF questionnaire. Thus, the real differences in ED symptoms between participants are reduced, which also negatively affects the correlations computed with this variable. Second, this study used self-reported data about searching topics on the Internet, but these answers that are based on participants’ recall might not be reliable and hence a more objective measure of the exact frequency of visiting certain websites is recommended. Furthermore, a check-list with topics was created for the purpose of this study but was not yet validated. In addition, with these measurement tools we did not distinguish between individuals who search topics in a “healthy” way and those who might be overly concerned and searched for this information in a compulsive fashion. Further studies should aim to tackle an anxiety-followed online-searching behavior, which would presumably be a better indicator of ED symptoms than seeking for information about food, diets, etc. in general. Finally, another important limitation of this study is the one-time data collection, which does not provide the researchers with information about the causational relationship between the variables. By using this methodology, it is unclear whether ED symptomatology induced more frequent online searching, or that searching about ED-related topics consequently provoked ED symptoms and perhaps contributed to their severity. Therefore, future studies should develop a longitudinal methodology in the area of EDs and online searching behavior in order to determine the direction of the causal relationship between these concepts.

## 5. Conclusions

The current study presents a novel finding in the research of EDs and behavior on the Internet. A greater BMI index and elevated maladaptive perfectionism might contribute to eating disorder-related behavior on the Internet through the experience of concerns relating to eating. Since nowadays the online world plays a great role in everyday life, it is reasonable to assume that individuals who have some symptoms of ED will more likely visit certain websites. Our study showed that, even though women in general search about nutrition, exercise or physical appearance on the Internet as well, it seems that those with a higher level of ED symptoms might be more persistent in finding such information. Therefore, further research about the reasons, as well as impacts, of searching for these topics on the Internet is warranted.

## Figures and Tables

**Table 1:** Descriptive statistics and Person’s correlation coefficients between the main variables of the study (*n* = 228).

	(1)	(2)	(3)	(4)	(5)	(6)	(7)
SCOFF	-						
Standards	−0.09	-					
Discrepancy	0.34 ***	0.17 **	-				
Order	−0.01	0.24 ***	−0.10	-			
Online searching	0.46 ***	−0.12	0.17 **	−0.01	-		
BMI	0.40 ***	−0.21	0.01	−0.01	0.26 ***	-	
Age	0.10	−0.23 ***	−0.12	0.04	0.16 *	0.36 ***	-
M	1.43	37.43	40.72	21.40	10.92	23.36	30.5
SD	1.39	6.67	16.64	4.89	3.75	4.25	9.43

Note: SCOFF – Sick, Control, One stone, Fat, Food; BMI – Body Mass Index; M – Mean; SD – Standard Deviation. * *p* < 0.05; ** *p* < 0.01; *** *p* < 0.001.

**Table 2:** Results of hierarchical regression analysis with online searching about eating disorder-related topics as the criterion (*n* = 228).

	Model 1			Model 2			Model 3		
	B	SE	β	B	SE	β	B	SE	β
Age	0.03	0.03	0.08	0.04	0.03	0.10	0.04	0.03	0.108
BMI	0.21	0.06	0.23 **	0.18	0.06	0.21 **	0.05	0.06	0.05
Standards				−0.05	0.04	−0.09	−0.03	0.04	−0.05
Discrepancy				0.05	0.02	0.21 **	0.02	0.02	0.07
Order				0.02	0.05	0.03	0.01	0.05	0.01
SCOFF							1.10	0.19	0.40 ***
R	0.27			0.34			0.49		
R^2^	0.08			0.12			0.24		
∆R^2^				0.04			0.12		
∆F	8.94 ***			3.50 *			33.44 ***		

Note: * *p* < 0.05; ** *p* < 0.01; *** *p* < 0.001.

**Table 3:** Results of the mediation analysis using SCOFF (Sick, Control, One stone, Fat, Food) result as a mediator between Discrepancy and online searching; BMI and online searching (*n* = 228).

Predictors		Coefficient	t-Values	Bootstrap 95% CI
Discrepancy	Total effect	0.04	2.66 **	
	Direct effect	0.01	0.35	
	Indirect effect	0.03		(0.02; 0.05)
BMI	Total effect	0.23	4.10 ***	
	Direct effect	0.08	1.47	
	Indirect effect	0.15		(0.10; 0.21)

Note: ** *p* < 0.01; *** *p* < 0.001.
